# Low-damage mechanised harvesting of shallots: from biomechanical interaction mechanisms to bionic and flexible design strategies

**DOI:** 10.3389/fpls.2026.1870450

**Published:** 2026-09-10

**Authors:** Honglei Zhang, Xiaoying He, Zhong Tang

**Affiliations:** School of Agricultural Engineering, Jiangsu University, Zhenjiang, China

**Keywords:** biomechanics, bionic drag reduction, flexible clamping, low-damage harvesting, shallot

## Abstract

With the rapid expansion of the shallot industrial scale, the high costs and low efficiency associated with traditional manual harvesting have emerged as core bottlenecks restricting the sustainable development of the industry. When deployed in shallot fields, existing general-purpose root-crop harvesting equipment encounters a typical engineering mechanics contradiction: the “high pull-out resistance of deep root systems” versus the “high vulnerability of tender pseudostems”. Consequently, achieving efficient, intact, and non-destructive harvesting remains challenging. To address the technical difficulties of the low-damage mechanised harvesting of shallots, this review systematically summarises the research progression of core engineering strategies based on the microscopic “machine-soil-plant” interaction mechanism. First, the viscoelastic rheological characteristics of shallot pseudostems and the pull-out mechanical responses of root-soil complexes are analysed to establish the physical damage boundaries for mechanical operations. Secondly, the bionic drag reduction technologies for soil-engaging components are highlighted, evaluating the efficacy of macroscopic geometric bionics and microscopic non-smooth surface features in optimising cutting stress fields and severing interfacial adhesion networks. Furthermore, the evolution of compliant mechanisms in the non-destructive clamping and conveying of pseudostems is explored. This includes an in-depth analysis of adaptive clamping mechanisms, which are transitioning from passive rigid-flexible coupling towards morphological enveloping driven by soft actuators. Finally, addressing the current wear-resistance bottlenecks of bionic materials in complex adhesive environments and the dynamic adaptation limitations of rigid-flexible coupled systems, this paper proposes cutting-edge developmental trends. These encompass the integration of multi-body dynamics and discrete element method (MBD-DEM) coupled simulations, alongside machine vision-based intelligent closed-loop control. This review aims to provide rigorous theoretical support and system integration references for the research and development of a new generation of intelligent, low-damage shallot harvesting equipment.

## 1 Introduction

Shallots (*Allium ascalonicum* L.) hold a significant position in the condiment processing and fresh markets due to their specific nutritional and agronomic value (Raeisi et al., 2016; Phaiphan et al., 2019; Moldovan et al., 2022). Following adjustments in agricultural industrial structures, shallot cultivation has expanded steadily in Asia, establishing it as an important economic crop for regional agricultural development (Raga et al., 2024; Apriyana et al., 2026; Zhang et al., 2026). However, the contradiction between expanding planting scales and the structural shortage of agricultural labour is increasingly prominent. Because shallot plants are densely packed and their root systems penetrate deeply into the soil, the harvesting process heavily relies on high-intensity manual labour. Statistical data indicate that manual harvesting is inefficient and constitutes a major proportion of the total field production cost, thereby severely restricting the profit margins of the industry (Kong et al., 2023). Therefore, advancing the mechanised upgrade of shallot harvesting equipment is an objective engineering requirement to ensure the sustainable development of the industry.

Despite the urgent demand for mechanised harvesting, the specific biomechanical properties of shallots and the complex farmland habitats have set a very high engineering threshold for equipment R&D. On the one hand, shallot pseudostems (the white part) are highly vulnerable tender and have high moisture content, with structural strengths significantly lower than those of conventional root crops such as potatoes and onions, making them highly susceptible to extrusion damage or cell rupture during mechanised clamping and conveying (Bao et al., 2021; Sahu et al., 2025; Shen et al., 2026) On the other hand, to ensure the integrity of whole-plant harvesting for commercial shallots, harvesting equipment must overcome the substantial pull-out resistance generated by the intertwining of deep root systems and heavy soil during extraction. The “low-damage requirement for pseudostems” and “high-load operation for root-soil interactions” constitute a significant engineering mechanics contradiction, causing existing general-purpose root-crop harvesters to easily induce severe mechanical damage during shallot field operations, making them difficult to adapt effectively.

In the specific context of this review, “low-damage” harvesting is defined as the optimization of mechanical interactions within the “machine-soil-plant” system to prevent both macroscopic physical fracture (such as pseudostem snapping or root-bulb tearing) and microscopic cellular crushing. Mechanically, a low-damage process requires that the external loads applied during digging, extraction, and clamping remain strictly below the viscoelastic yield limit of the plant tissues. Agronomically, this structural preservation is mandatory to maintain the morphological integrity, commercial grade, and post-harvest shelf-life of the shallots. Reviewing existing root-crop harvesting technologies, scholars at home and abroad have developed integrated harvesting systems for crops such as carrots (Zhou et al., 2023b; Senthilkumar et al., 2024), garlic (Min et al., 2025; Hou et al., 2025) onions (Min et al., 2020; Jung et al., 2024; Rathinakumari et al., 2024), and leeks (Feng et al., 2023), which combine digging, cleaning, and collection functions. For example, Zhang X. L. et al. (2023) significantly reduced the damage rate of handheld garlic harvesters by optimising digging and clamping parameters; Dorokhov et al. (2023) also effectively controlled physical loss during the cleaning and separation process of onions through variable-angle conveyor systems. However, translating these technologies to shallot harvesting often results in failure.

This fundamental failure to directly translate technology stems from the vast biological and agronomic differences between shallots and other cited crops. Unlike the highly lignified stems of cassava or the robust, thick-skinned harvestable bulbs of onions and garlic, the primary harvestable organ of a shallot heavily incorporates its extremely tender pseudostem. From an anatomical perspective, shallot pseudostems possess a significantly lower viscoelastic yield limit, making their cellular structures highly susceptible to irreversible rupture under clamping forces that would otherwise be safe for leeks or garlic. Agronomically, shallots are cultivated with a much higher planting density, resulting in a complex, densely intertwined root architecture and modified local soil compaction conditions. Consequently, while the basic cutting and pulling mechanisms derived from potatoes or carrots provide a valuable theoretical framework, the actual failure mechanisms of shallots are uniquely sensitive. The safe operational force windows—specifically the required pulling, cutting, and clamping forces—must be drastically recalibrated to accommodate their fragile pseudostem anatomy and the severe interfacial soil adhesion typical of shallot habitats.

A dynamic vibration analysis of shallot field harvesters by Nguyen et al. (2025) clearly pointed out that the high-frequency amplitudes generated by traditional rigid digging heads during the extraction phase are the core source of damage inducing fractures in pseudostems and bulbs. Furthermore, when dealing with high-humidity and heavy soil habitats, rigid soil-engaging components not only consume excessive power but are also prone to causing additional secondary drag damage to fragile pseudostems due to severe interfacial adhesion effects. This demonstrates that traditional rigid, high-power operation modes are fundamentally unable to accommodate the extremely sensitive biomechanical vulnerability of shallots.

To resolve the aforementioned physical conflicts, modern agricultural equipment research is accelerating its evolution from simple rigid optimisation of mechanical structures to interdisciplinary fields involving “bionic drag reduction” and “compliant mechanisms”. By reconstructing soil-engaging components through simulating the digging morphology and non-smooth surface characteristics of soil animals, it is expected that harvesting power consumption can be significantly reduced from the dimensions of macroscopic stress fields and microscopic interfacial inhibition. Meanwhile, the introduction of flexible clamping technology based on soft robotics provides a feasible path for achieving non-destructive morphological enveloping of irregular and fragile plants.

To ensure terminological rigor and eliminate conceptual ambiguity within this interdisciplinary review, it is necessary to explicitly differentiate the advanced engineering mechanisms discussed. Biomimetic refers to the direct geometric replication of biological structures (e.g., extracting multi-toed claw contours), whereas bionic denotes the broader functional integration of these biological principles into engineering systems (e.g., achieving bionic drag reduction). Regarding load adaptation during mechanical interactions, flexible describes the physical property of passive materials (such as elastomers or springs) capable of elastic deformation under stress to disperse contact pressure. Compliant mechanisms refer to the engineered kinematic structures that transfer forces and generate motion through designed elastic deformation rather than traditional rigid hinges. Finally, soft-robotic mechanisms represent active, pneumatically- or hydraulically-driven continuous actuators constructed from hyperelastic materials, specifically engineered to spontaneously achieve non-linear morphological enveloping around complex target geometries.

To systematically capture these interdisciplinary advancements, a rigorous literature search and screening protocol was implemented primarily using the Web of Science (Core Collection) database. The search strategy employed combinations of keywords including “shallot” or “Allium crops”, alongside “mechanised harvesting”, “low-damage”, “biomechanics”, “bionic drag reduction”, and “flexible clamping”. During the screening phase, studies exclusively focused on pure agronomic practices were excluded, prioritizing literature that explicitly addressed the core physical conflict between high soil pull-out resistance and pseudostem vulnerability. Finally, the selected high-quality literature was systematically classified into the core thematic areas discussed below for in-depth analysis.

In view of this, this paper systematically reviews the latest research progression in the field of low-damage mechanised shallot harvesting: first, the biomechanical characteristics of shallot pseudostems and the machine-soil-plant interaction mechanism are analysed to define the boundaries of mechanical damage; second, the efficacy of bionic drag reduction technology in the optimisation of soil-engaging components is evaluated, as well as the evolutionary direction of flexible adaptive clamping mechanisms in non-destructive conveying; finally, the application potential of Multi-Body Dynamics and Discrete Element Method (MBD-DEM) coupling simulation technology and intelligent closed-loop control in future equipment R&D is discussed prospectively, aiming to provide rigorous theoretical support and a reference for the system integration of a new generation of intelligent, low-damage shallot harvesting equipment.

## 2 Biomechanical mechanisms and constitutive modelling in the harvesting process of *Allium* crops

Revealing the physical and mechanical interaction mechanisms of the “machine-soil-plant” system is the theoretical prerequisite for the design and system optimisation of low-damage shallot harvesting equipment. As a typical viscoelastic material, the sensitivity of shallots to external loads is influenced not only by genetic characteristics but also by the physiological state of the tissues and the operational environment. By quantifying their mechanical response patterns, these critical yield points and fundamental properties serve as the essential physical inputs for establishing high-fidelity simulated coupling models (e.g., MBD-DEM). These simulated coupling models effectively replace traditional static constitutive analysis, providing dynamic and explicit physical boundary constraints for the design of drag-reducing soil-engaging components and adaptive flexible clamping mechanisms.

### 2.1 Rheological properties and damage yield boundaries of pseudostem tissues

Research indicates that the macroscopic mechanical damage boundaries of *Allium* crops are primarily constrained by their own biological morphological characteristics. Significant differences exist in epidermal strength among different varieties, and the material properties of the plant surface directly determine their angle of repose and static friction coefficient (Kurniawan et al., 2019). Specifically, the elastic modulus is a paramount parameter characterizing the stiffness and the viscoelastic yield limit of the pseudostem; it fundamentally dictates the maximum allowable clamping force that can be applied before irreversible cellular rupture occurs. Concurrently, the friction coefficient—encompassing both the internal friction between plant fibers and the external kinetic friction between the plant epidermis and mechanical end-effectors—determines the slippage behavior and the vulnerability to abrasive surface damage during high-speed grasping and conveying. A precise calibration of both the elastic modulus and the friction coefficient is indispensable for establishing the safe operational force windows required by adaptive flexible clamping mechanisms.

Although varieties and morphology lead to fluctuations in macroscopic mechanical parameters, the internal tissues of the shallot pseudostem exhibit high homogeneity, and the differences in mechanical properties across different anatomical regions are not significant (Yang et al., 2022). This characteristic simplifies the establishment of an equivalent force model for the whole plant, making mechanical simulations based on unified parameters feasible. Regarding tissue micro-mechanical properties, the subcellular-scale strength of the middle lamella in the epidermis of *Allium* crops under tensile loading is significantly higher than that of cell wall fragments, with greater connection strength at the cell corners (Zamil et al., 2013). This provides a microscopic perspective for understanding the tissue tearing mechanisms of pseudostems under macroscopic tension.

In terms of dynamic load responses, the loading rate and contact mode are the core parameters determining the rheological characteristics and yield boundaries of pseudostems (Han et al., 2023). Under external loads, the stress concentration and rupture limits of pseudostems are highly dependent on the angle and geometric morphology of the contact tools. The shear force, bio-yield force, and their corresponding deformations under different states exhibit significant non-linear evolution (Yilmaz and Gökduman, 2024). Furthermore, research on cutting failure limits has further indicated a distinct positive correlation between cutting resistance and crop neck diameter (Jin et al., 2024).

**Figure 1:**
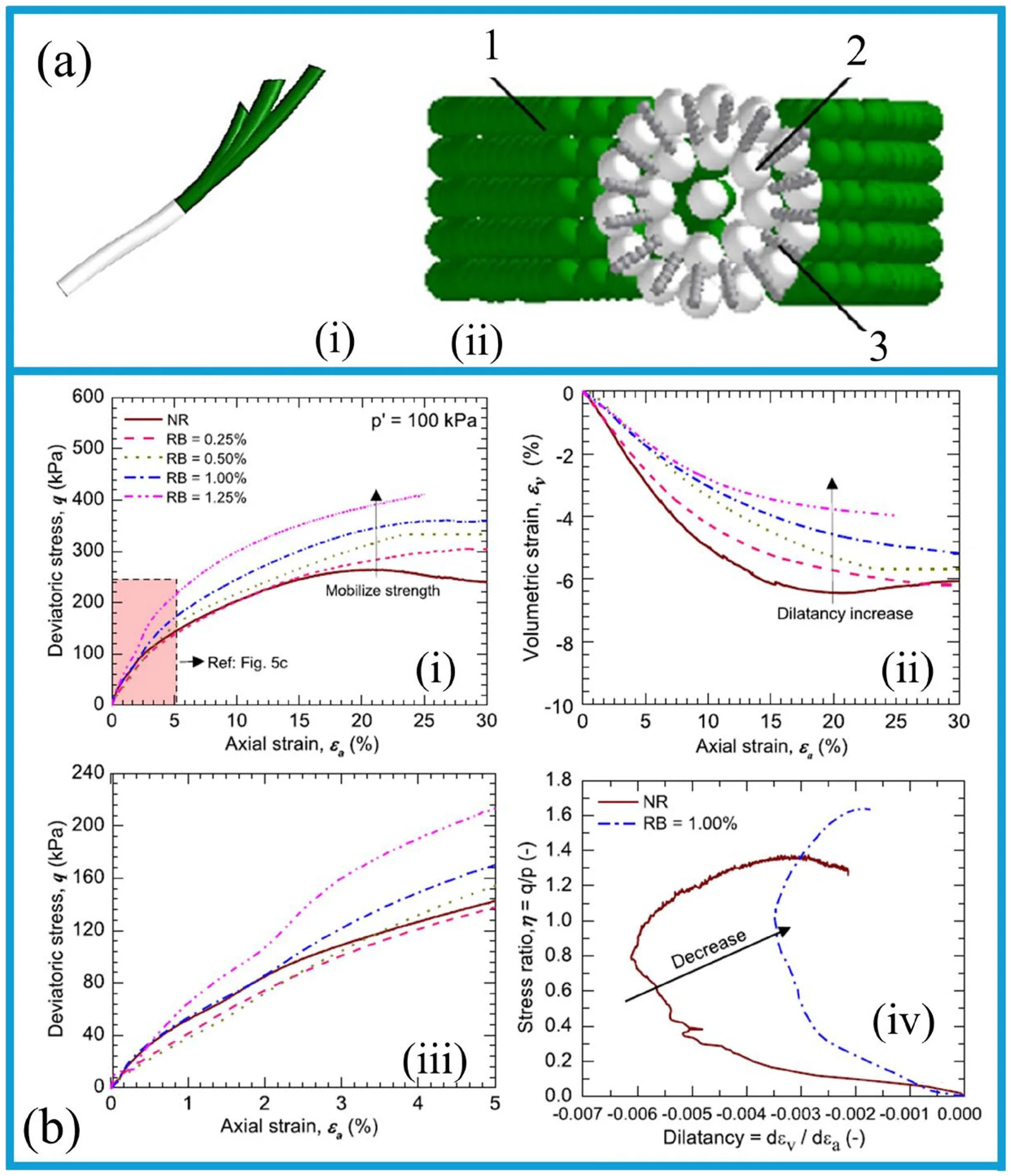
Microscopic modelling of shallot plants and mechanical response characteristics of the root-soil complex: **(a)** Three-dimensional geometric configuration of shallot seedlings and their discrete element method (DEM) equivalent representation (1-leaf particle domain, 2-pseudostem particle domain, 3-root system particle domain) (Zhang et al., 2025); **(b)** Intervention curves of different root biomasses (RB) on the triaxial drained shear stress-strain response of the root-soil complex under a confining pressure of 100 kPa (Alam et al., 2022).

To accurately replicate this complex dynamic contact process, researchers introduced the Hertz-Mindlin contact theory to construct a high-fidelity discrete element method (DEM) mechanical model of shallot seedlings (see **Figure 1a**) (Zhang et al., 2025). By finely dividing the plant into independent particle domains such as leaves, pseudostems, and root systems, and verifying their stress-strain characteristics under actual compressive loading experiments, key mechanical parameters of *Allium* plants, including normal/tangential contact stiffness, ultimate stress, and Poisson’s ratio, were accurately calibrated. The acquisition of these quasi-static and dynamic characteristics has laid a solid data foundation for setting the parameter domains of mechanical forces.

The aforementioned fundamental physical parameters directly drive the subsequent establishment of mechanical boundaries for the mechanisms. Addressing the issue that the clamping process is highly prone to inducing extrusion damage, a rheological constitutive model derived from the linear viscoelastic characteristics of tender vegetables can systematically analyse the dynamic boundaries between elastic clamping force and clamping spacing (Jin et al., 2024). Research verifies that by matching the optimal combination of spring stiffness and clamping time, the plastic damage deformation of the pseudostem can be minimised. In summary, the mechanical damage boundary of the shallot pseudostem is the result of deep interactions between morphological parameters and dynamic operational parameters; establishing a constitutive model based on viscoelastic rheology is the theoretical basis for achieving low-damage clamping.

It is important to explicitly acknowledge that the current literature suffers from a significant scarcity of biomechanical parameters specifically quantified for shallots (Allium ascalonicum). Consequently, much of the foundational mechanical data discussed relies on homologous Allium crops, such as onions, leeks, and garlic. While utilizing data from these related crops is justified by their shared family-level cellular architectures and generic viscoelastic rheological trends, their absolute mechanical values cannot be treated as perfectly interchangeable. Shallots possess distinctly higher slenderness ratios, specific moisture contents, and unique tissue densities compared to robust onion bulbs or highly fibrous leek stems. Therefore, the lack of a dedicated, high-precision biomechanical database for shallot pseudostems represents a critical research gap. Future empirical studies must prioritize quantifying shallot-specific mechanical thresholds—such as precise elastic modulus, Poisson’s ratio, and ultimate yield stress—to calibrate more accurate and reliable equipment design parameters.

### 2.2 Pull-out response and critical state mechanisms of the root-soil complex

During the extraction phase of shallots, the pull-out mechanical strength of the root-soil complex, formed by the intertwining of the root system and the surrounding soil medium, is a key physical quantity determining the harvesting power consumption and the whole-plant intactness rate (Han et al., 2023). Through its spatial reinforcement effect, the root system significantly alters the shear strength and cohesion of the soil, while the moisture content, adhesiveness, and compactness of the soil constitute the physical constraint boundaries for root detachment. Therefore, an in-depth analysis of the stress field evolution, interfacial failure mechanisms, and critical state characteristics of the root-soil complex during dynamic loading is the mechanical prerequisite for achieving efficient and low-damage extraction (Ma et al., 2020; Ao et al., 2025).

Existing mechanical characterisation methods primarily define the constitutive features of the complex through advanced geotechnical tests. Results from triaxial drained shear tests indicate that the mechanical response of the root-soil complex is significantly influenced by root biomass (RB) (Alam et al., 2022). Under low confining pressure, the presence of roots enhances the deformation resistance of the soil, and its stress-strain behaviour exhibits a distinct strengthening effect with the increase in biomass (see **Figure 1b**). In addition to biomass, the geometric configuration of the root system also contributes significantly differently to the strength of the complex; the differing soil remoulding effects of root systems with various spatial layouts directly imply the importance of adapting the digging trajectory to the root growth polarity (Ji and Science, 2019).

Analysed from a macroscopic mechanical framework, critical state soil mechanics theory provides an important criterion for evaluating the failure process of the root-soil interface. The concept of the soil critical state defines an equilibrium state where stress and strain tend to remain constant during the shearing process, which holds theoretical guiding significance for assessing the degree of soil fragmentation during harvesting. On this basis, the critical state behaviour of unsaturated silty soil is further modulated by hysteresis effects (Estabragh et al., 2020), and the fines content in the soil also exerts a unique shifting impact on the critical state line (Ekinci et al., 2020). These research findings on multi-factor coupling have laid a theoretical foundation for establishing a constitutive model of the root-soil complex that encompasses complex habitat factors.

Regarding the dynamic adjustment of pull-out parameters, the evolution pattern of extraction resistance is deeply regulated by operating speed and geometric vectors. Focusing on the extraction trajectory post-digging, comparisons of the performance of subsoiling shovels with different geometric configurations reveal that specific configurations (e.g., L-shaped shovels) possess significant mechanical superiority in reducing pull-out resistance and inhibiting physical damage to roots and stems (Yang W. et al., 2024). To further analyse the separation mechanism between soil clods and crops at a microscopic level, Kang et al. (Long et al., 2025) introduced the Tavares UFRJ breakage model to characterise the interactive behaviour between *Fritillaria thunbergii* bulbs and soil aggregates, revealing the microscopic impact of vibration parameters on soil disturbance and the depth distribution of bulb particles.

Addressing the anchorage characteristics of *Allium* crops, quantitative research has confirmed the functional relationship among pull-out force, root curve shape, root length, and soil strength (Ennos, 1990). To more accurately simulate interfacial failure during the extraction process, academia has introduced elastic softening constitutive laws and non-linear damage models; by considering strength degradation and root fracture modes during interfacial shearing, a high-fidelity description of complex interfacial interactions has been achieved (Zhu et al., 2022). Furthermore, root volume density has been established as a core morphological indicator determining the internal friction angle and cohesion of the soil (Forster et al., 2022). In summary, the pull-out mechanical response of shallots is the result of the coupled effect of root morphological parameters and critical soil characteristics; future theoretical research should focus on developing non-linear damage models capable of characterising the dynamic field extraction resistance in real time.

Quantitatively, the engineering contradiction of shallot harvesting can be defined by a critical mechanical inequality. For a successful, non-destructive extraction, the dynamic extraction force (*F_ext_*) exerted by the harvesting machinery must overcome the total root-soil pull-out resistance (*F_soil_*) without exceeding the ultimate yield limit (*F_yield_*) of the pseudostem tissue. This relationship is governed by the condition: $F_{soil}<F_{ext}\leq \frac{F_{yield}}{\gamma }$, where γ represents the dynamic safety coefficient accounting for field vibrations and instantaneous impact loads.

In conventional operations, particularly within highly cohesive and high-humidity soils, *F_soil_* increases significantly due to capillary adhesion and soil compaction. Consequently, the required extraction force often exceeds the allowable mechanical limit of the tender tissues (*F_soil_* > *F_yield_*). When this physical boundary is breached, macroscopic fracture or microscopic cellular crushing is inevitable. Therefore, the core objective of modern harvesting engineering is twofold: reducing *F_soil_* through bionic drag-reduction and soil-loosening technologies, and maximizing the allowable *F_ext_* by optimizing stress distribution via adaptive flexible clamping.

### 2.3 Coupling simulation mechanisms of the discrete element method and multi-body dynamics

Although traditional macroscopic mechanical tests can obtain the fundamental physical parameters of shallot pseudostems and the root-soil complex, they struggle to dynamically replicate the instantaneous, non-linear microscopic contact interaction processes among the “machine-soil-plant” system during harvesting. With the rapid development of computational mechanics and computer simulation technologies, the coupled application of Multi-Body Dynamics (MBD) and the Discrete Element Method (DEM) has provided a novel research paradigm for revealing the cutting disturbance mechanisms of soil-engaging components and the dynamic clamping damage mechanisms of compliant mechanisms (Liu W. J. et al., 2024; Fang et al., 2025). By constructing high-fidelity virtual physical models, the characteristics of stress distribution and the evolution rules of microscopic force chains can be intuitively extracted, thereby significantly reducing the trial-and-error costs of physical prototypes and shortening the research and development cycle (Zhou et al., 2023a; Lu et al., 2024).

**Figure 2:**
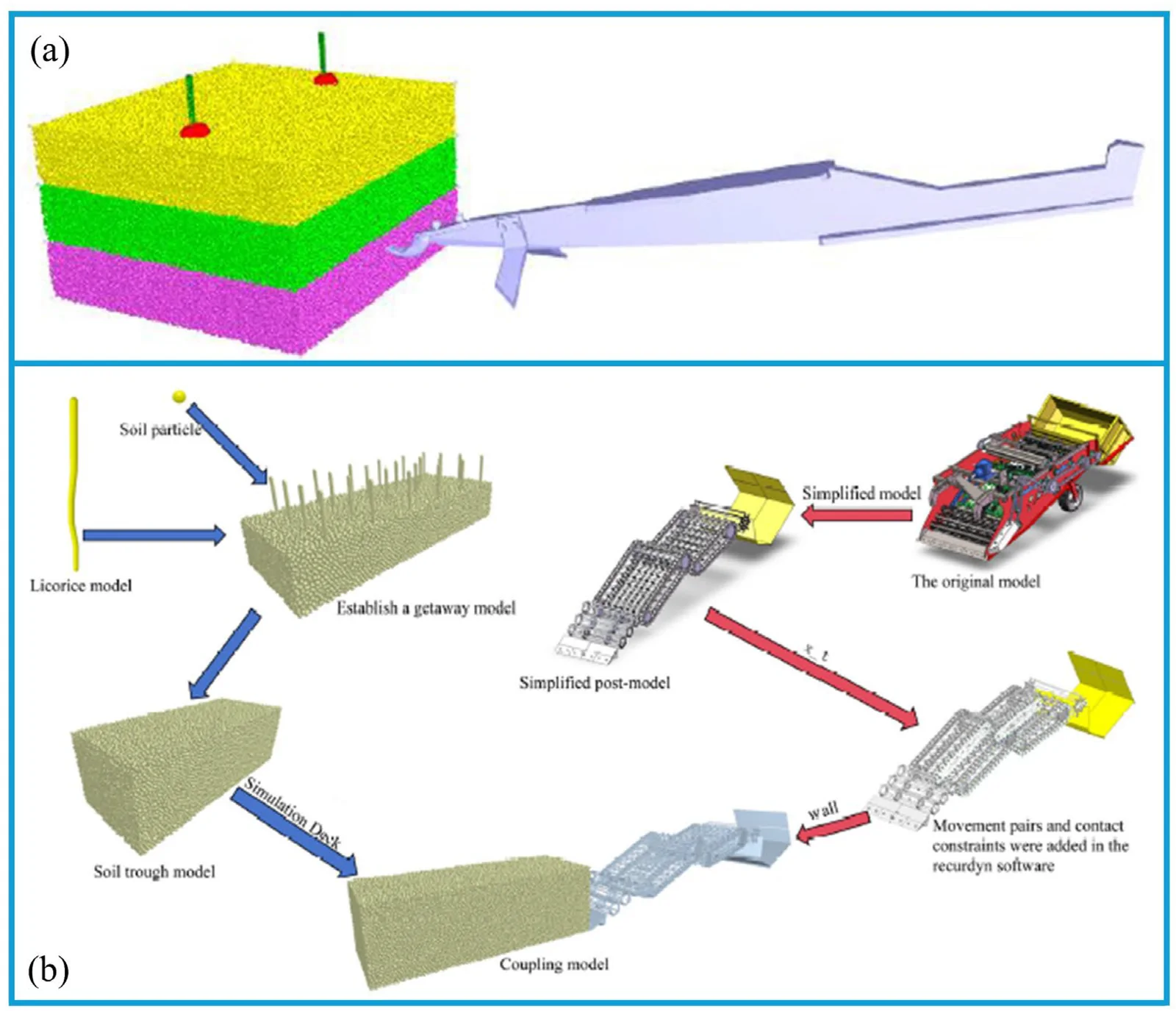
Discrete element method and multi-body dynamics numerical simulation modelling of the mechanised shallot harvesting process: **(a)** Construction of the virtual soil bin model and setting of discrete element parameters (Zhou et al., 2023a); **(b)** Simulation modelling workflow for the machine-plant-soil interaction (Lu et al., 2024).

In simulating the interactions between soil-engaging components, soil, and root systems, the discrete element method has demonstrated unique advantages in analysing granular mechanics (Ding et al., 2022; Wang et al., 2022; Chen et al., 2023; El-Emam et al., 2023; Liang et al., 2023). By constructing a virtual soil bin model and setting precise contact parameters (see **Figure 2a**), the variation patterns of the displacement field of soil particles under the action of soil-engaging components can be deeply analysed, confirming the reliability of numerical simulations in optimising the geometric parameters of digging components (Zhou et al., 2023a). In the simulation of complex operational conditions, a full-process harvesting simulation model established using the DEM-MBD coupling method can detail the complete dynamic response from digging to conveying (see **Figure 2b**), thereby accurately analysing the motion trajectories and velocity distributions of roots and stems on the elevating separation device (Lu et al., 2024). Focusing on the vibratory digging process, collaborative simulation analysis using EDEM-RecurDyn can clarify the non-linear mapping relationships among vibration frequency, penetration angle, and digging resistance, further revealing the drag reduction mechanism of the vibratory mechanism (Li et al., 2026).

Furthermore, bionic digging shovels developed by drawing on the geometric characteristics of biological organisms have been verified through coupled simulations to exhibit notable drag reduction and soil-crushing efficacy in specific soil environments (e.g., latosol). Experimental data indicate that they possess significant mechanical advantages over traditional structures (Yang R. B. et al., 2024). In the quantitative analysis of the complex contact between agricultural machinery components and granular materials, discrete element technology can similarly explore the dynamic interaction mechanisms of guiding components, demonstrating exceptionally high technical superiority (Liang et al., 2022).

Focusing on the contact collision and clamping damage mechanisms of tender crops, rigid-flexible coupling and viscoelastic constitutive models provide critical analytical tools (Yan et al., 2018b; Yan et al., 2018a; Liu J. Z. et al., 2019; Yan et al., 2019; Pi et al., 2021; Song et al., 2024). By establishing a viscoelastic-plastic contact model, the degree of deformation and the evolution rules of stress distribution in tuber crops during transient collisions can be accurately predicted (Liang et al., 2023). In the field of flexible clamping, Han M. et al. (2024) constructed a rigid-flexible coupled simulation model of a plant stem clamping device based on ADAMS dynamics software, dynamically analysing the force boundaries and response characteristics of the clamping system during operation. This exploration breaks the limitations of traditional static measurements and provides an important reference for the kinematic optimisation of non-destructive clamping mechanisms for shallot pseudostems. To further enhance the high fidelity of the models, a discrete element model of shallot seedlings established based on the Hertz-Mindlin with Bonding contact model calibrated the normal/tangential contact stiffness and ultimate stress of the seedlings through Plackett-Burman experiments, laying the foundation for precise simulations (Zhang et al., 2025).

With the deepening of research, multi-physics field coupling technology is gradually becoming a frontier trend in the mechanistic study of modern agricultural machinery equipment. For example, applying DEM-CFD coupling technology to the simulation of complex flow fields and granular particle separation in harvesters can achieve high-precision tracking of the dynamic evolution process of multiphase flows (Ding et al., 2022; El-Emam et al., 2023). Although current research on whole-plant rigid-flexible coupled simulations for *Allium* crops is still in its infancy, the aforementioned coupling technologies have already demonstrated immense potential in the mechanical modelling of related agricultural crops. In the future, there is an urgent need to construct high-fidelity digital twin models encompassing the “bionic digging shovel-high humidity heavy soil-whole shallot plant” system, to deeply analyse the fracture damage mechanisms under actual field conditions.

## 3 Bionic drag reduction mechanisms of soil-engaging components and interfacial detachment technologies

During the field operations of agricultural machinery, soil-engaging components inevitably need to overcome substantial soil cutting resistance and adhesive friction. For crops like shallots, which possess deep root systems and extremely fragile pseudostems, excessive pull-out resistance not only increases power consumption but also translates into strong dragging loads on the plants, thereby inducing the mechanical fracture of the pseudostems. If modern root-crop harvesting equipment lacks targeted drag reduction and separation designs, it will be difficult to control physical losses while ensuring operational efficiency (Dorokhov et al., 2023). In recent years, the complex application of bionic principles in the field of agricultural engineering has provided a novel approach for the optimal design of soil-engaging components. By extracting the excellent macroscopic geometric morphology and microscopic non-smooth surface characteristics of soil animals in nature, bionic components can systematically reconstruct the dynamic “machine-soil” contact interaction mechanism from two physical dimensions: macroscopic cutting stress field optimisation and microscopic interfacial adhesion inhibition.

### 3.1 Macroscopic geometric bionics and cutting stress field optimisation mechanisms

Macroscopic geometric bionics primarily focuses on soil-dwelling animals with highly efficient digging capabilities (e.g., pangolins, mole crickets, badgers, mole rats, etc.) (Xu et al., 2016; Lv et al., 2018; Zhang et al., 2020; Hu et al., 2023; Tian et al., 2023; Ma et al., 2024). Long-term natural evolution has enabled the claws, toes, or body surface scales of these organisms to evolve unique spatial curved configurations and multi-dimensional non-linear contours. Introducing these into the design of soil-engaging components can fundamentally alter the macroscopic failure modes of the soil during mechanical penetration, transforming the traditional single rigid extrusion damage into low-energy composite shear and tensile damage, thereby significantly homogenising and optimising the cutting stress field around the soil-engaging components.

**Figure 3:**
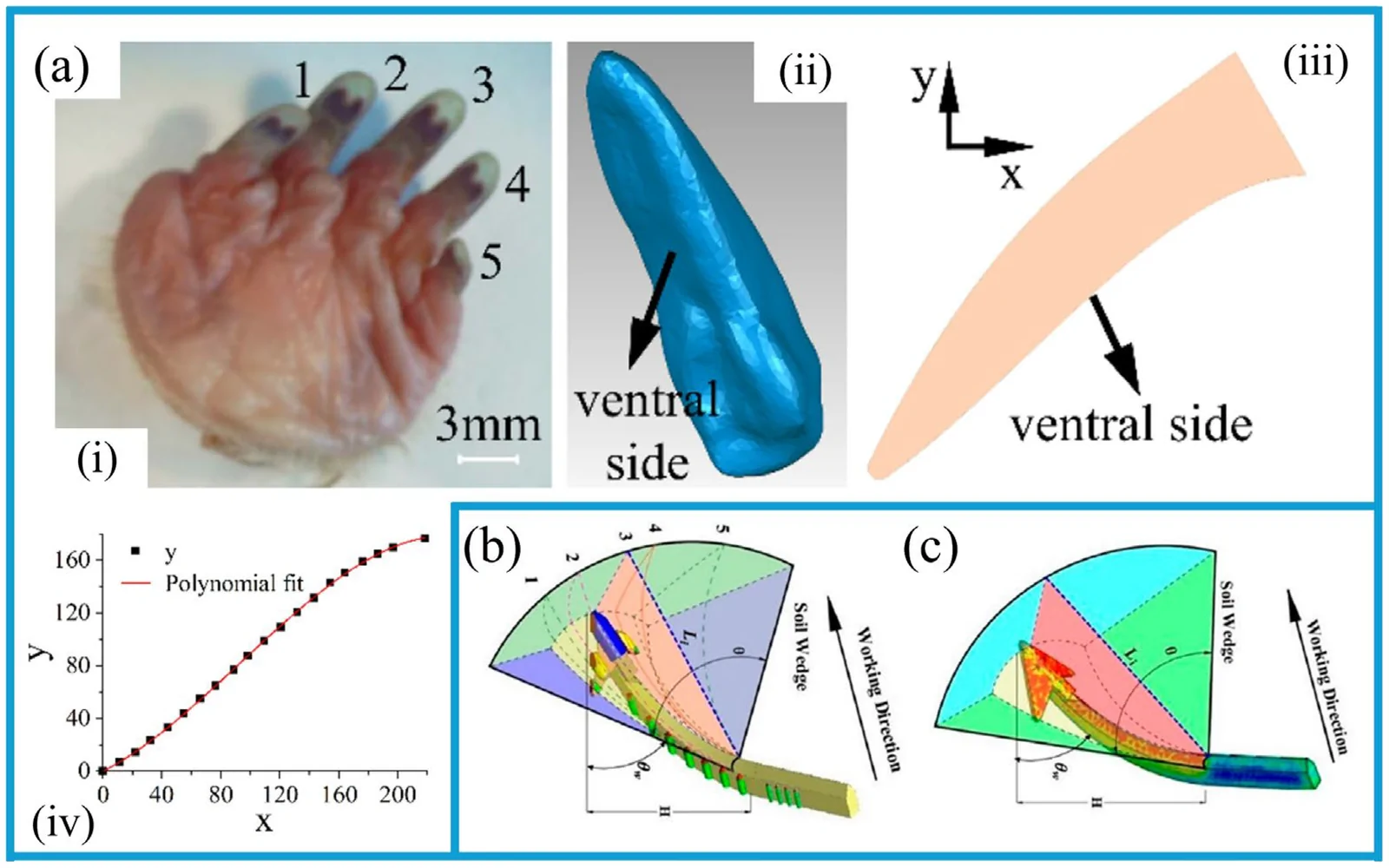
Extraction of macroscopic morphological bionic features of soil-engaging components and optimisation of the cutting stress field: **(a)** The extraction of macroscopic morphological features of the mole rat’s front claw and the three-dimensional reconstruction workflow (including the ventral morphology of the left front claw, the 3D mesh model, the mid-sagittal plane section, and the fitting curve of the contour coordinate points for the 4th digit) (Yu et al., 2022); **(b)** Fitting curve of the contour coordinate points and dynamic mechanical analysis; **(c)** Comparison of the soil failure models and dynamic mechanical responses under the cutting action of the traditional standard subsoiling shovel and the bionic subsoiling shovel (Song et al., 2025).

In the contour reconstruction of digging teeth and subsoiling shovels, the claw characteristics of multi-toed animals have demonstrated notable drag reduction potential. By extracting the mid-sagittal plane contour coordinates of a mole rat’s digit using reverse engineering technology and performing high-precision curve fitting (see **Figure 3a**), the developed bionic digging shovel can significantly reduce traction resistance under specific conditions where the penetration angle is greater than 30°, achieving a maximum drag reduction rate of 13.33% (Yu et al., 2022). From the perspective of the dynamic mechanism of stress field optimisation, the bionic structure based on the mole rat claw configuration can effectively guide the forward soil to generate composite shear and tensile failure (see **Figure 3b**). Compared to the large-area compaction and extrusion caused by traditional standard subsoiling shovels, its maximum stress is substantially reduced by 52.96% (Song et al., 2025). This physical characteristic provides a crucial macroscopic drag reduction mechanism reference for crops like shallots, which require deep subsoiling and are extremely intolerant of forceful dragging.

Addressing the specific harvesting requirements of various deep-rooted crops, the geometric characteristics of various digging organs have been widely translated into engineering applications. Drag-reducing shovels and rotary tillage blades designed using the badger claw toe as a prototype have effectively overcome the bottlenecks of insufficient soil disturbance and excessive cutting resistance in traditional machinery by optimising the curvature of the soil-cutting surface (Guan et al., 2022; Zhou et al., 2023a; Zhou et al., 2023a). Furthermore, bionic digging devices developed by extracting the contours of rabbit’s front paws and prairie mole rat claws have demonstrated significant mechanical penetration and soil-crushing efficacy in complex habitats such as latosol (Xiaopeng et al., 2019; Yang R. B. et al., 2024; Wang Z. et al., 2025). Additionally, features such as the concave curved surface of an ostrich toenail (Zhang et al., 2019), the morphological combination of the front claws of anteaters and American badgers (Akter and Basak, 2022), and the three-dimensional geometric structure of the mole cricket’s foreleg digging claw (Zhang et al., 2021) have all been proven capable of achieving soil penetration and drag reduction in different soil environments by reshaping the stress gradient of the cutting surface.

Beyond digging claws, the streamlined contours and special scale structures of biological surfaces have also provided abundant morphological inspiration for the homogenisation of cutting stress fields. Non-linear bionic curved surfaces constructed by extracting the contour of pangolin scales using reverse engineering (Jia et al., 2018a), simulating the streamlined features of a catfish head (Yang et al., 2025), and incorporating the rib patterns on the surface of a Hawaiian shellfish (Feng et al., 2019), have all exhibited significant drag reduction effects in heavy soil environments. Composite bionic designs (such as integrating the curve characteristics of an earthworm’s head and a pangolin’s claw toe) further confirm that the “point contact” mode generated by corrugated structures can effectively weaken the shear strength of the soil continuum (Liu G. M. et al., 2024).

At the more microscopic level of cutting and penetration mechanisms, the sharp contours of biological mandibles and incisors likewise possess extremely high value for engineering translation. Bionic cutters designed by extracting the biomechanical morphology of locust mandibles and bamboo rat lower incisors have significantly optimised the cutting performance of crop fibres (Su et al., 2024). The mathematical equation fitting methods for these edge curves provide solid methodological support for the edge sharpening and non-linear optimisation of shallot digging shovels.

In summary, macroscopic geometric bionics effectively reduces the penetration resistance during mechanical advancement by altering the penetration angle, the curvature of the soil-cutting surface, and the non-linear characteristics of the cutting edge of soil-engaging components, thereby reshaping the initial stress distribution at the soil interface. However, relying solely on the reconstruction of macroscopic geometric contours remains insufficient to completely eliminate the physical adhesion phenomenon of soil particles at the metal interface when confronting high-humidity and heavy soils. Therefore, to break through the bottleneck of high interfacial friction, it is necessary to further integrate microscopic-scale non-smooth surface detachment technologies.

### 3.2 Microscopic non-smooth surface morphology and interfacial detachment effects

When harvesting shallots in habitats with high moisture content or heavy soil, continuous water films and soil compaction layers are highly prone to forming on the surfaces of soil-engaging components, thereby inducing severe interfacial adhesion effects. This physical adhesion at the microscopic level not only significantly increases the traction resistance of the advancing agricultural machinery but also destroys the natural fluidity of the soil, subsequently imposing destructive additional dragging stress on the extremely fragile pseudostems (Yang et al., 2023; Wang et al., 2024; Zhang C. et al., 2024). Organisms in nature that have long inhabited heavy soil environments (such as dung beetles and earthworms) have, through evolution, developed non-smooth body surface morphologies characterised by convex domes, concave pits, and ripples. These microscopic topographies can effectively sever the capillary adhesion networks at the interface, trap air to form micro air cushions, and substantially reduce the actual contact area. This fundamentally alters the boundary conditions of the friction interface from the underlying physical mechanisms, providing a core mechanical basis for the detachment and drag reduction design of soil-engaging components.

The non-smooth structures represented by the body surface of dung beetles serve as typical morphological prototypes for anti-adhesion designs; the microscopic geometric features of convex domes and concave pits distributed on their body surfaces demonstrate substantial interfacial inhibition capabilities (Dacke et al., 2014). In engineering translation, composite bionic non-smooth surfaces constructed by superimposing the surface characteristics of different organisms (such as the micro-spined convex domes of crayfish and the microscopic scales of sandfish) can not only thoroughly disrupt the soil’s capillary adhesion interface at the microscopic level but also significantly increase the average disturbance velocity of local particles by 57.8%, demonstrating excellent microscopic detachment and soil-crushing efficacy (Wang XY. et al., 2025). Analysed from a mechanical mechanism perspective, convex dome structures can induce microscopic stress concentrations at the contact surface, breaking the soil continuum and effectively reducing sliding friction resistance; whereas concave pit structures significantly inhibit normal adhesion forces by altering the motion trajectories of local soil particles. By utilising advanced materials such as ultra-high molecular weight polyethylene (UHMW-PE) to process bionic soil-engaging surfaces, and by quantifying characteristic parameters such as the height-to-diameter ratio of the convex domes, the dual superiority of non-smooth surfaces in enhancing microscopic soil disturbance and reducing interfacial adhesion forces has been further verified (Soni et al., 2007; Massah et al., 2021).

**Figure 4:**
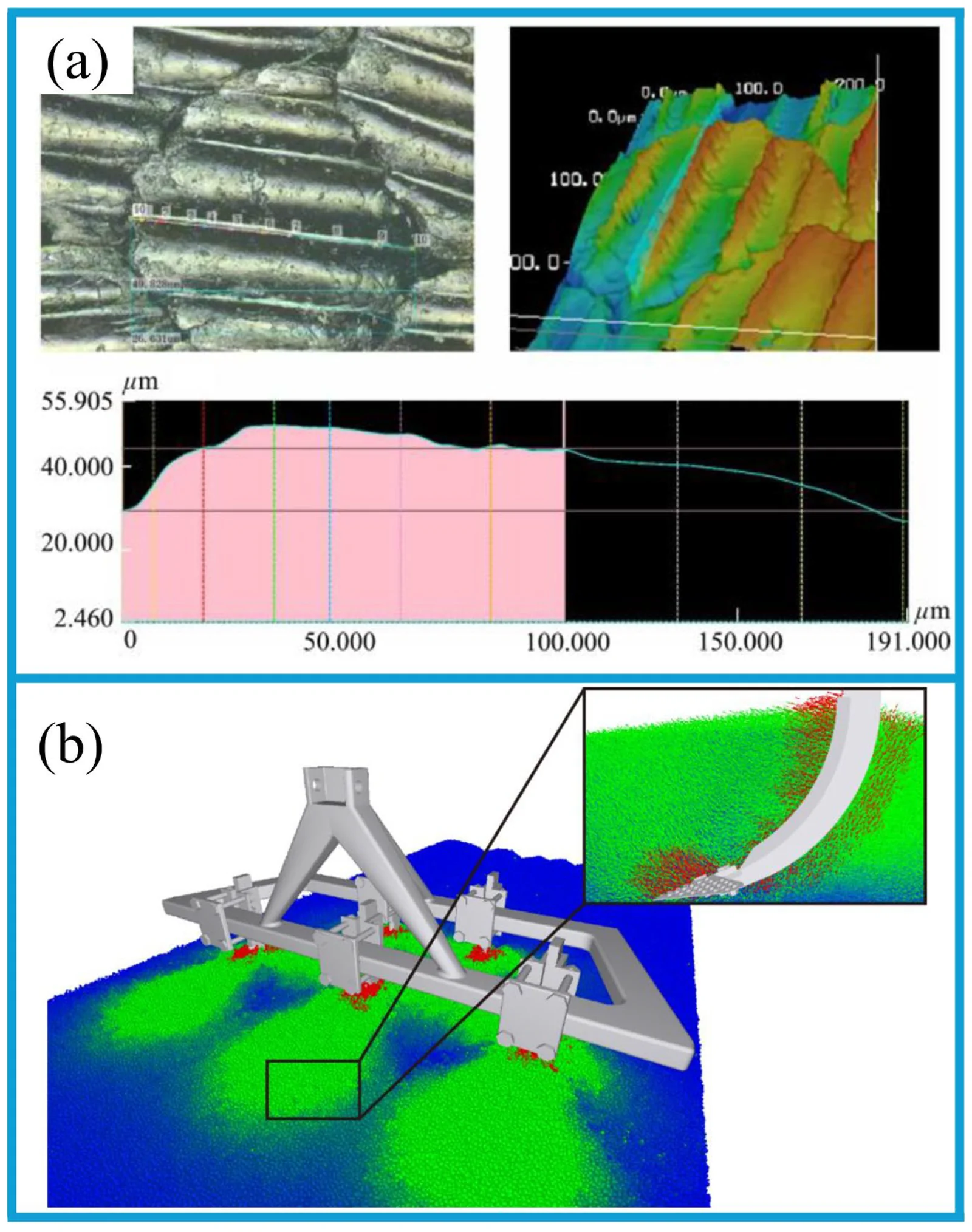
Microscopic non-smooth morphological features of soil-engaging components and machine-soil interaction drag reduction mechanisms: **(a)** Microscopic geometric morphology of hammerhead shark skin placoid scales in the transverse direction (observed via laser scanning confocal microscopy, LSCM) (Niu et al., 2022); **(b)** Velocity distribution map of soil particles during composite bionic subsoiling operations (Wang X. Y. et al., 2025).

In addition to terrestrial soil animals, the body surface microstructures of aquatic organisms likewise provide important cross-habitat inspirations for interfacial anti-adhesion. Characterisation results of the microscopic morphology of the transverse cross-section of shark skin placoid scales using laser scanning confocal microscopy (LSCM) reveal (see **Figure 4a**) that their unique discontinuous ridge-like geometric configurations can effectively block the direct contact networks between soil particles and mechanical surfaces (Niu et al., 2022). By physically severing the continuous interfacial water film, this structure significantly weakens the normal adhesion force, supplementing crucial fluid dynamics rationales for the detachment design of soil-engaging components in shallot harvesting machinery when tackling high-humidity and viscous habitats.

To further break through the drag reduction limits of single-morphology bionics, the research focus is gradually shifting towards a coupled drag reduction strategy of “morphological bionics + interfacial lubrication”. Bionic coupled interfaces manufactured by extracting the non-linear curves of the earthworm body surface and integrating self-lubricating properties have verified the synergistic drag reduction effects of multi-dimensional physical characteristics (Liu G. et al., 2019). Furthermore, composite bionic furrow openers simulating the multiple characteristics of earthworms have not only reduced operational resistance but also effectively improved the physicochemical structure of the surrounding probed soil (Jia et al., 2018b). This design philosophy of multi-dimensional decoupling and composite superposition indicates that microscopic detachment technology is advancing towards higher-order, system-level mechanical-electrical-hydraulic synergistic evolution.

To systematically illustrate the translation of these biological mechanisms into agricultural engineering, the typical bionic prototypes, their extracted morphological features, and their corresponding engineering applications in soil-engaging components are summarized in **Table 1**.

**Table 1:** Summary of biological prototypes and their biomimetic applications in agricultural soil-engaging components.

Biological prototype	Bionic category	Extracted morphological feature	Engineering application	References
Mole rat/Badger	Macroscopic Geometric	Multi-toed claw contours and spatial curved surfaces	Subsoiling shovels, rotary tillage blades	(Yu et al., 2022; Zhou et al., 2023a) (Feng et al., 2019; Guan et al., 2022; Zhou et al., 2023a; Song et al., 2025)
Pangolin/Catfish	Macroscopic Geometric	Streamlined scale contours and point-contact structures	Bionic curved digging surfaces	(Jia et al., 2018a; Feng et al., 2019; Liu G. M. et al., 2024; Yang et al., 2025)
Locust/Bamboo rat	Macroscopic Geometric	Non-linear sharp curves of mandibles and incisors	High-efficiency crop fibre cutters	(Hu et al., 2023; Su et al., 2024)
Dung beetle/Earthworm	Microscopic Non-smooth	Convex domes, concave pits, and surface ripples	Anti-adhesion interfaces, furrow openers	(Dacke et al., 2014; Liu G. et al., 2019) (Jia et al., 2018b)
Shark/Sandfish	Microscopic Non-smooth	Discontinuous ridge-like placoid scales, micro-spines	Interfacial detachment and micro-disturbance structures	(Niu et al., 2022; Wang X. Y. et al., 2025)

### 3.3 Performance adaptation evaluation of bionic structures in complex habitats

Although macroscopic geometric bionics and microscopic non-smooth surface technologies have demonstrated significant drag reduction performance in controlled soil bin tests and finite element simulations, they still face severe physical adaptability challenges when mapped directly to the actual field habitats of mechanised shallot harvesting. High-yield shallot cultivation areas typically maintain high soil water-holding capacities, constituting a typical “high-humidity and highly cohesive” physical environment. Under such non-ideal boundary conditions, the detachment efficacy of bionic soil-engaging components is highly susceptible to degradation.

The primary mechanical bottleneck lies in the failure of microscopic topological structures caused by abrasive wear. Field soils are generally rich in high-hardness abrasive particles. During continuous high-load cutting processes, the microscopic non-smooth morphologies on the surfaces of mechanical components are highly prone to micro-cutting and fatigue wear. For example, the fidelity of the microscopic geometry of bionic surfaces processed using anti-adhesion materials such as ultra-high molecular weight polyethylene (UHMW-PE) is difficult to maintain over the long term under high-intensity field friction (Soni et al., 2007; Guan et al., 2021; Ma et al., 2022). Once non-smooth morphologies such as convex domes or concave pits are worn flat, the micro air cushion effect trapping air at the interface will completely collapse, leading to a rapid rebound in soil capillary adhesion forces.

To break through this lifespan bottleneck, reconstructing the operational shovel tip by utilising the self-protection mechanisms of microscopic geometries (such as extracting the microstructural characteristics of the dung beetle clypeus) has become an important method to extend the effective operational cycle of anti-adhesion features (Wang et al., 2023). Concurrently, the introduction of composite bionic surfaces further optimises the dynamic detachment mechanisms. Numerical simulation analyses indicate that composite microscopic textures can significantly alter the motion states of interfacial soil particles, substantially increasing the average disturbance velocity of the particles by 57.8% (see **Figure 4b**) (Wang X. Y. et al., 2025). This physical enhancement of local particle fluidity not only effectively tears apart the capillary adhesion networks but also further reduces macroscopic operational energy consumption, demonstrating excellent microscopic detachment and soil-crushing capabilities.

Secondly, the wide spatio-temporal fluctuations in field soil moisture content impose stringent constraints on the stability of non-smooth surfaces (Chandio et al., 2021; Yin et al., 2024). When soil-engaging components penetrate deeply into high-humidity and heavy soil layers, excessive free water is highly prone to filling the microscopic crevices of the non-smooth surfaces, forming a dense “mud-wrapping” phenomenon, which leads to a sharp increase in interfacial friction resistance. Relevant applicability evaluations have also confirmed that single-morphology bionic textures struggle to accommodate a wide range of viscous rheological working conditions, and are highly susceptible to completely losing their detachment function when the water film thickens (Massah et al., 2021).

Furthermore, the dense fibrous root networks of *Allium* crops profoundly alter the soil rheological parameters within local microhabitats. In this state, bionic soil-engaging components no longer cut a single medium, but rather an anisotropic complex possessing high shear strength (Han and Liu, 2018; Mao et al., 2020; Han DL. et al., 2024). This necessitates that future bionic soil-engaging designs must transcend the limitations of purely mechanical structures, incorporate advanced surface engineering technologies to enhance underlying wear resistance, and introduce mechanical-electrical-hydraulic multi-source sensing systems to achieve high-frequency adaptive active closed-loop control. This will thereby ensure a high degree of physical adaptability for microscopic detachment mechanisms under complex habitat constraints.

## 4 Adaptive clamping mechanisms and low-damage conveying control strategies for tender pseudostems

In the operational chain of mechanised shallot harvesting, clamping and extraction is a key procedure that directly acts upon the economic parts of the crop. Because shallot pseudostems possess mechanical properties such as high moisture content, abundant thin-walled cells, and a low rheological yield limit, traditional root-crop harvesting machinery equipped with rigid clamping chains or constant-pressure operational structures is highly prone to generating local stress concentrations on the crop surface, thereby inducing cell rupture and mechanical abrasion. To achieve precise control of contact stress whilst ensuring sufficient pull-out force, the end-effectors of harvesting equipment must evolve towards “compliant mechanisms” (Guan et al., 2021). In recent years, the optimisation of passive rigid-flexible coupled clamping mechanisms and the introduction of pneumatic/hydraulic soft actuators have provided forward-looking mechanical solutions for realising the adaptive clamping and coordinated conveying of tender crops (Ahmad et al., 2021; Yue et al., 2022).

Furthermore, transitioning this theoretical low-damage framework into practical application requires explicit consideration of field variability, which imposes highly dynamic boundary conditions on the harvesting system. Soil moisture and soil type directly govern the interfacial adhesion and cohesive strength, causing the required pull-out resistance to fluctuate drastically across different field plots. Simultaneously, variations in planting density and individual shallot size (e.g., pseudostem diameter and morphological curvature) demand that harvesting end-effectors possess robust spatial adaptability. Consequently, a truly effective low-damage harvesting framework cannot rely on static parameters; it must integrate adaptive drag-reduction geometries to mitigate variable soil impedance, alongside compliant grasping mechanisms capable of non-linearly enveloping varying crop sizes without causing adjacent interference in densely planted rows.

### 4.1 Stress dispersion characteristics of passive flexible clamping interfaces

Passive flexible clamping mechanisms primarily absorb excess kinetic energy through the elastic deformation of materials by covering the surfaces of rigid actuators with highly elastic materials (e.g., sponge, rubber) or connecting them in series with mechanical springs, dynamically increasing the actual contact area between the actuators and the crop surface (Chen et al., 2022; Zhou et al., 2022; Du C. X. et al., 2024; Lin et al., 2025; Yu et al., 2025). According to classical contact mechanics theory, under the premise of a constant total clamping load, an expansion of the contact area can exponentially reduce the local maximum compressive stress, thereby transforming concentrated loads into uniformly distributed surface loads and effectively delaying the occurrence of tissue damage (Huang et al., 2021).

**Figure 5:**
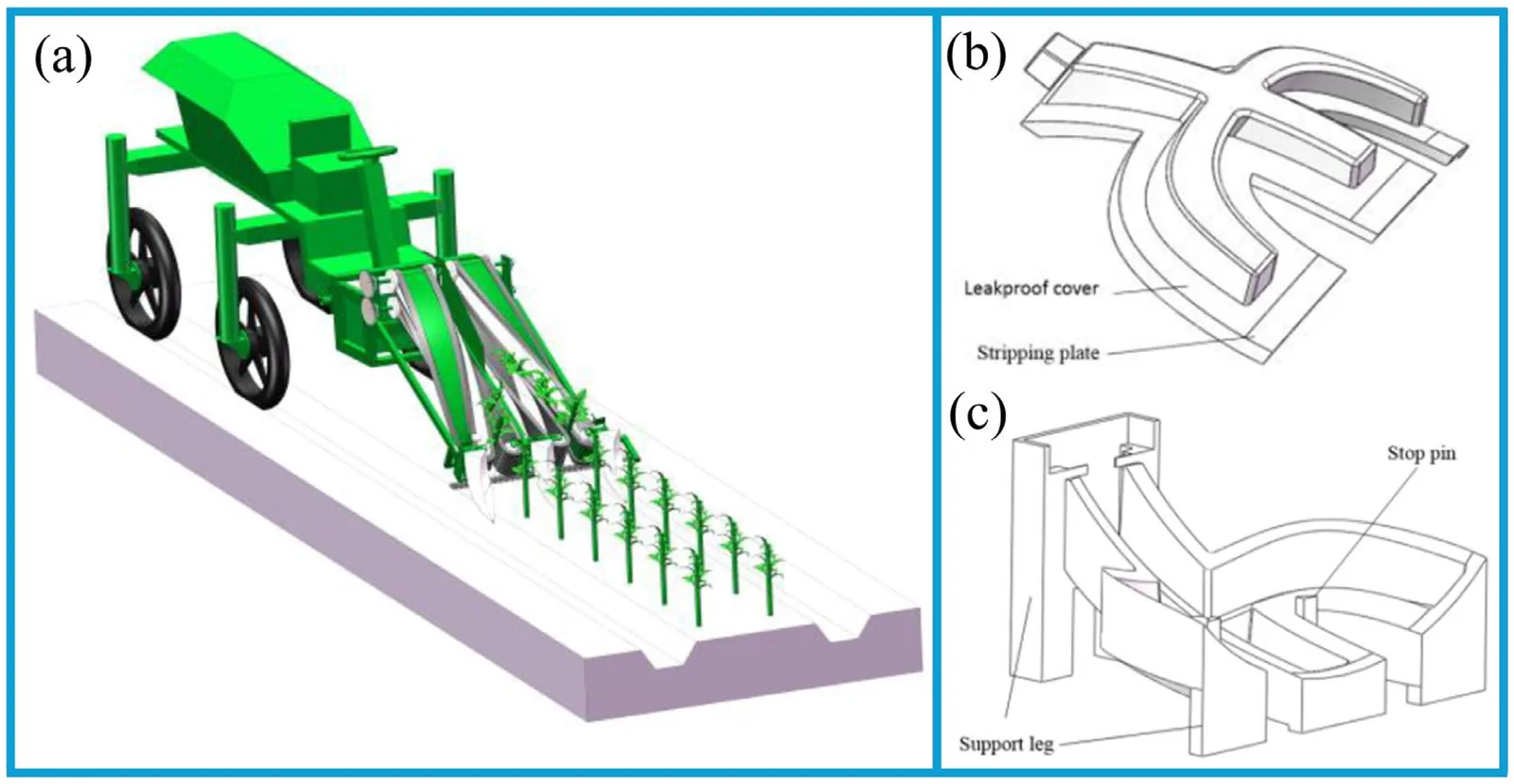
Schematic of flexible clamping harvesting operations and mould design for bionic soft actuators: **(a)** Schematic diagram of the field operation process of the flexible clamping harvester for oilseed rape stalks (Xiong et al., 2023); **(b)** Design drawing of the upper mould for the casting of the bionic soft actuator (conjoined muscle structure); **(c)** Design drawing of the lower mould for the casting of the bionic soft actuator (conjoined muscle structure) (Te et al., 2020).

Research on the optimisation of clamping parameters for tender crops indicates that elastic interfaces are significantly effective in dispersing contact stress (Cui et al., 2018; Qing Y. R. et al., 2021; Zhang F. et al., 2023). For example, during the harvesting process of oilseed rape stalks, by optimising the clearance and compressive force of the clamping belts (see **Figure 5a**), it is possible to maintain efficient conveying whilst controlling the damage rate at a low level of 5.22% (Xiong et al., 2023). Addressing the unique physical properties of shallots, a clamping device with flexible protrusions was developed by calibrating the normal/tangential contact stiffness and optimal stress using the Hertz-Mindlin with Bonding contact model. It has been verified that this device can further reduce the mechanical damage rate to between 2.76% and 3% by leveraging the stress dispersion effect of the flexible discs (Zhang et al., 2025).

Because leafy vegetable crops possess significant viscoelastic characteristics, the evolution of plastic damage during the clamping process is essentially a time-dependent rheological process. Rheological constitutive models constructed based on linear viscoelasticity theory can quantitatively describe the regularities of plastic deformation induced by mechanical clamping through mathematical equations (Jin et al., 2024). Research verifies that the synergistic matching of spring stiffness combinations (e.g., 2.0 N/mm and 0.6 N/mm) with the clamping time window has a decisive impact on tissue creep deformation. By accurately setting the stiffness of flexible elements, stress fluctuations at the contact interface can be effectively suppressed, achieving the minimisation of cumulative plastic damage.

Furthermore, the mechanical properties of crops often exhibit a dynamic evolutionary trend after detaching from the soil habitat. External airflow disturbances or post-harvest water loss can both lead to changes in plant tissue density, which requires the pre-tightening force setting of the clamping interface to possess extremely high physical robustness (Xing et al., 2021; Hou et al., 2023). In summary, passive flexible clamping achieves effective stress dispersion within specific ranges through spring resistance adjustment or elastomer facing. However, when confronted with excessively large coefficients of variation in field plant diameters or extremely irregular cross-sectional shapes, passive materials often struggle to achieve omnidirectional morphological enveloping. Once the amount of extrusion breaches the elastic limit, the underlying rigid structure may still cause physical interference. Therefore, clamping technology must inevitably iterate towards soft actuators that possess higher degrees of freedom and can actively conform to target contours.

### 4.2 Non-destructive enveloping and grasping mechanisms of soft actuators

As a core branch of soft robotics, soft actuators demonstrate significant safety advantages in grasping tender and fragile targets due to their significant compliance, representing a core evolutionary direction for intelligent harvesting equipment (Qin et al., 2017). These actuators are typically constructed from hyperelastic materials, and their core physical characteristic lies in their ability to spontaneously generate large-scale non-linear deformations according to the actual contours of the targets, thereby achieving “morphological enveloping”. This continuum deformation mechanism fundamentally circumvents the local stress concentrations induced by rigid contact, providing underlying technical support for the non-destructive harvesting of shallot pseudostems.

**Figure 6:**
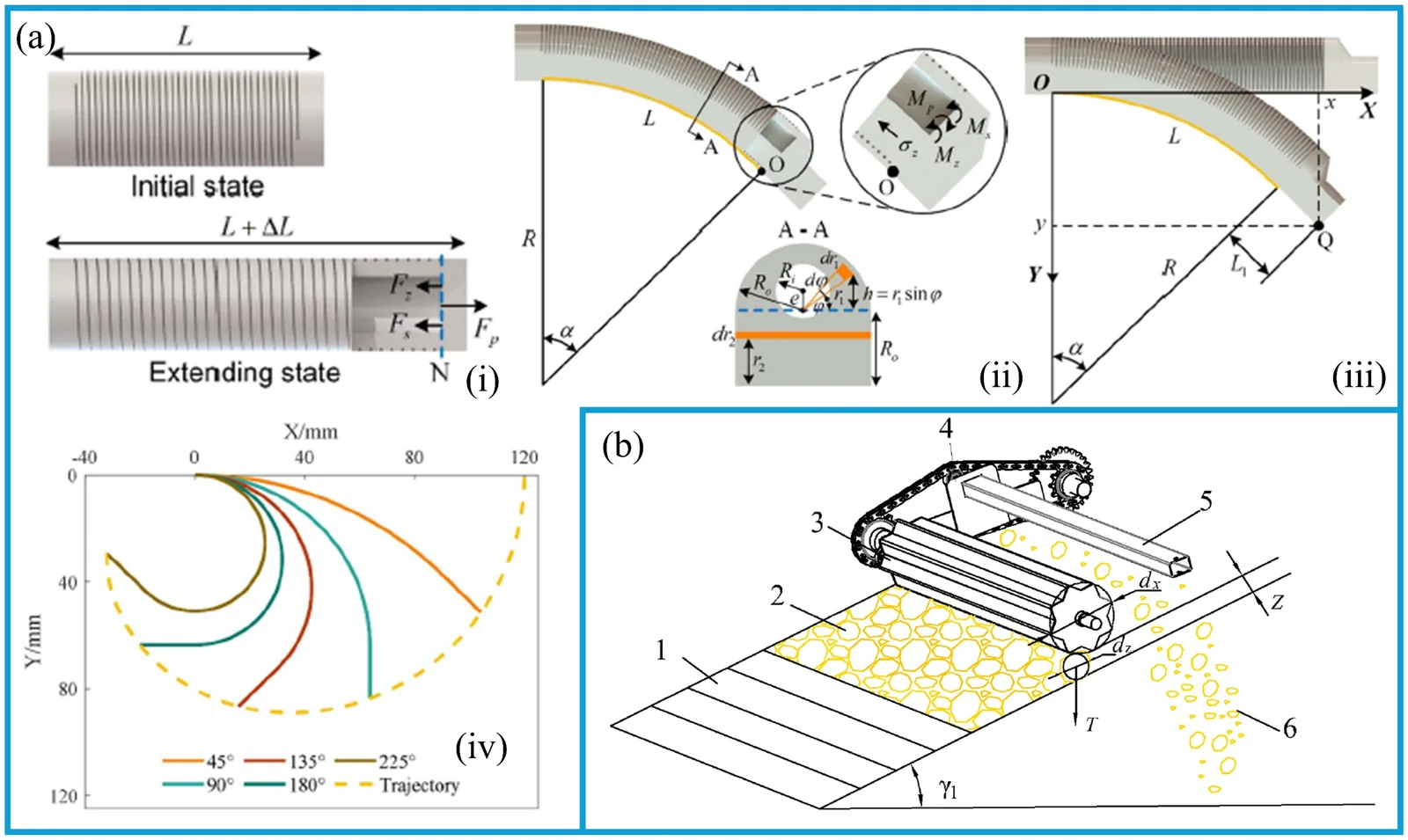
Schematic of mechanism modelling for soft actuators and the conveying, soil-crushing, and separation mechanism: **(a)** Static model, bending kinematic model, and characterisation of the actuator bending posture and fingertip motion trajectory of soft extending and bending actuators (Cheng et al., 2023); **(b)** Structural layout of the soil-crushing device in the digging system prototype (1-conveying device, 2-large soil clod, 3-soil-crushing roller, 4-connecting plate, 5-support frame, 6-crushed soil particles) (Du J. P. et al., 2024).

Regarding the dynamic adaptation of grasping postures and mechanism modelling, the kinematic and static characteristics of soft actuators determine the distribution quality of their contact forces. By constructing non-linear force models of the actuators, the deformation characteristics of soft extending and bending actuators can be systematically analysed, and the motion trajectories of the fingertips can be accurately predicted (see **Figure 6a**) (Cheng et al., 2023). This physical mechanism-based predictive capability enables the clamping system to automatically adjust its enveloping morphology based on target position and posture feedback, ensuring the maintenance of an optimal contact force vector distribution when grasping targets with complex geometric shapes. For example, in the research and development of a soft actuator simulating the locomotion mechanism of jellyfish, precise mould casting processes ensure the physical consistency between internal pneumatic cavities and conjoined muscle structures (see **Figures 5b, c**) (Te et al., 2020), thereby providing structural guarantees for large-scale enveloping deformations. Furthermore, introducing bionic designs based on pneumatic superstructures (e.g., inspired by the *Mimosa* pulvinus) can maintain extremely high grasping stability on complex contact interfaces through innovative driving modes (Zhang W. H. et al., 2024).

Innovations in driving mechanisms and the parametrised description of structures are crucial for achieving precise harvesting. Modularly architected diaphragm-type pneumatic soft grippers can achieve adaptive adjustments according to the morphological characteristics of crops with different diameters (Navas et al., 2021b; Navas et al., 2021a). To achieve ultimate contact safety whilst ensuring sufficient grasping loads, academia has begun utilising Pythagorean Hodograph (PH) curves to parametrise the shape kinematics of soft fingers, combined with Euler-Bernoulli modelling to predict control inputs, thereby achieving optimal morphological enveloping and grasping when confronting extremely fragile targets such as mushrooms (Mbakop et al., 2023).

To further enhance operational robustness in unstructured field habitats, multi-bionic composite actuation technologies are gradually becoming research hotspots. A multi-bionic composite actuator integrating the grasping structure of an eagle claw, the friction-enhancing characteristics of a gecko foot, and the structural colour of a butterfly wing not only enhances grasping force on complex interfaces but also provides cutting-edge technical solutions for addressing environmental disturbances during field harvesting (Zhang et al., 2022). In summary, by actively conforming to the geometric morphology of targets, soft actuators physically circumvent the rupture of thin-walled cells in pseudostems caused by traditional rigid extrusion. This paradigm shift from “forceful constraint” to “flexible enveloping” is the core pathway to achieving high-fidelity shallot harvesting.

### 4.3 Damage inhibition and trajectory optimisation in the rigid-flexible coupled conveying process

During the combined harvesting of shallots, the dynamic conveying phase after the plants detach from the soil constraint is a high-incidence area for secondary mechanical damage. The damage mechanisms at this stage primarily originate from complex vibrations transmitted by the mechanical system and forced displacement stresses induced by mismatched motion trajectories. Research indicates that the large amplitudes and high accelerations generated by the harvesting mechanism during the initial operational stage are the primary factors inducing physical damage to the bulbs of *Allium* crops (Nguyen et al., 2025). Furthermore, the high-frequency vibrations of the mechanical system can also induce internal fatigue within the tender tissues, subsequently degrading the storage and transportation quality of commercial shallots (Faheem et al., 2021). Therefore, maintaining the dynamic stability of the operational process through kinematic optimisation and active control measures is crucial for inhibiting secondary damage.

The precise coordination of extraction and conveying trajectories is the mechanical foundation for eliminating additional stress. Establishing a kinematic speed ratio model between rotational speed and forward speed can effectively ensure the steady posture of plants during the conveying process, thereby eliminating forced traction stress caused by speed mismatch (Ji et al., 2022). By parametrically modelling and optimising the trajectories of key operational components, interference zones and abrupt speed change points in the motion curves can be identified and eliminated, reducing the risk of mechanical collisions from the kinematic source (Qing Y. et al., 2021). Under complex dynamic direction-changing conditions, adopting a rigid-flexible coupled design of multi-bar mechanisms can further achieve high-speed and smooth transitions of plant postures, suppressing transient impact loads (Yin et al., 2021).

To deeply analyse the stress evolution during the conveying process, Multi-Body Dynamics and Discrete Element Method (MBD-DEM) coupling simulation technology provides a high-fidelity analytical tool. By analysing the motion trajectories and velocity field distributions of the plants on the elevating separation chain, researchers can intuitively identify the physical nodes prone to inducing damage, thereby guiding the structural optimisation of low-damage separation devices (Lu et al., 2024). In actual mechanism design, addressing the soil-crushing and separation requirements of *Allium* crops, the development of dedicated soil-crushing devices and the realisation of their kinematic coordination with conveying mechanisms can reduce the risks of mechanical abrasion and fracture of roots and stems to an extremely low level (approximately 2.44%) whilst ensuring efficient soil crushing (Du J. P. et al., 2024).

Beyond the optimisation of static structural parameters, intelligent closed-loop regulation represents the frontier evolutionary direction for inhibiting dynamic damage. By introducing visual recognition technology into the conveying system to perceive the positional deviation and morphological distortion of targets in real time, and using this as a feedback signal to drive actuators for dynamic compensation, it can be ensured that shallots remain under protection whilst passing through unstructured conveying channels (Cheng et al., 2023). This coordinated control paradigm based on “dynamic visual perception-flexible active compensation” will provide a fundamental mechanical guarantee for completely eradicating the problem of secondary damage during the shallot harvesting process.

To systematically evaluate the engineering applicability and physical bottlenecks of the aforementioned technologies across different harvesting stages, a critical synthesis of the reviewed literature is presented in **Table 2**. This comparative analysis highlights the original crop systems from which these technologies were derived, their core biomechanical mechanisms, and the primary limitations that must be overcome for successful adaptation to low-damage shallot harvesting.

**Table 2:** Comparative synthesis of core technologies, originating systems, and main limitations for shallot harvesting adaptation.

Technology category	Original crop/system focus [ref]	Core mechanism/evidence category	Main limitations for shallot adaptation
Traditional Rigid Digging & Subsoiling	Cassava, Potatoes, General Root Crops (Dorokhov et al., 2023) (Yang W. et al., 2024)	Overcoming high bulk density soil resistance via sheer mechanical force and rigid trajectory optimization.	Induces high-frequency vibrations causing irreversible tissue rupture in tender shallot pseudostems and bulbs (Nguyen et al., 2025).
Macroscopic Geometric Bionics	Heavy soil habitats (e.g., badgers, mole rats) (Guan et al., 2022; Yu et al., 2022)	Reshaping cutting stress fields from single rigid extrusion to low-energy composite shear/tensile damage (Zhou et al., 2023a).	Fails to inhibit microscopic capillary adhesion networks when confronted with the high-humidity, sticky soil specific to shallot fields.
Microscopic Non-Smooth Surfaces	Earthworms, Dung beetles, Shark skin (Dacke et al., 2014; Jia et al., 2018b; Niu et al., 2022)	Breaking continuous water films and reducing actual contact area via micro-domes/pits (interfacial detachment) (Massah et al., 2021).	Micro-textures suffer rapid abrasive wear from high-hardness field particles; severe “mud-wrapping” occurs under extreme moisture (Wang et al., 2023; Yin et al., 2024).
Passive Flexible Clamping	Leafy vegetables, Oilseed rape stalks (Xiong et al., 2023; Jin et al., 2024)	Dispersing maximum compressive stress using elastic materials (sponges/springs) to delay plastic damage (Huang et al., 2021; Zhang F. et al., 2023).	Struggles to achieve full morphological enveloping for shallots with large diameter variations; underlying rigid interference persists.

### 4.4 Critical engineering synthesis and performance evaluation

To transcend the descriptive enumeration of individual bionic and compliant designs, it is essential to subject these mechanisms to a rigorous, criteria-driven comparative analysis. The transition from controlled laboratory environments to the complex dynamics of the shallot field requires evaluating these technologies against standardized engineering metrics. **Table 3** presents a semi-quantitative synthesis of the core technologies discussed in Sections 3 and 4, critically comparing their performance across critical operational criteria: efficiency, tribological durability (wear resistance), environmental adaptability, and technological maturity.

**Table 3:** Semi-quantitative engineering performance comparison of low-damage harvesting technologies.

Technology/mechanism	Drag-reduction/damage mitigation rate	Adaptability to high soil moisture	Tribological wear resistance	Operating speed/frequency	Technological maturity	Overall suitability for shallots
Macroscopic Geometric Bionics	Moderate-High (15-30% drag reduction)	Poor (Prone to mud accumulation)	High (Bulk material strength)	High (Compatible with tractor speeds)	High (Commercial viability)	Requires integration with active vibration; inadequate as a standalone solution in cohesive soils.
Micro-scale Non-smooth Surfaces	High (Up to 40% initial adhesion reduction)	Moderate (Capillary bridge disruption)	Low (Micro-textures degrade rapidly via abrasive soil wear)	High	Low-Moderate (Durability bottleneck)	High theoretical potential, but severely limited by tribological fatigue life in abrasive agricultural soils.
Passive Flexible Clamping (Sponges/Springs)	Moderate (Disperses peak compressive stress)	High (Independent of soil moisture)	Moderate (Material fatigue over continuous cycles)	Moderate-High	High (Widely used in vegetable harvesters)	Effective for average sizes; lacks robust non-linear morphological adaptation for varying shallot diameters.
Soft-Robotic Actuators (Fluidic/Pneumatic)	Exceptional (Near-zero cellular rupture)	High (Independent of soil mechanics)	Low-Moderate (Susceptible to puncture from sharp soil debris)	Low (Dynamic pneumatic response lag)	Low (Lab-scale prototyping)	Ultimate low-damage solution, but currently bottlenecked by slow actuation speeds and poor resilience to harsh field debris.

As synthesized in **Table 3**, while micro-scale non-smooth surfaces exhibit excellent theoretical drag reduction, their practical application is severely constrained by low tribological wear resistance against abrasive soil particles. Conversely, although soft-robotic actuators provide the most optimal damage mitigation by respecting the pseudostem’s viscoelastic boundaries, their slow kinematic response and vulnerability to mechanical puncture render them insufficiently mature for high-speed field operations. Therefore, the immediate future of shallot harvesting machinery relies on hybrid systems: macroscopic geometric subsoilers combined with highly durable passive flexible clamping mechanisms, balancing damage mitigation with the rigorous demands of agricultural durability.

## 5 Conclusions and future prospects

### 5.1 Main research conclusions

As a vegetable crop with extremely high economic added value, the mechanised harvesting of shallots has long been constrained by the physical conflict between “high pull-out resistance” and the “extreme vulnerability of pseudostems”. Starting from the mechanical mechanisms of the microscopic “machine-soil-plant” interaction, this paper systematically reviews the key engineering strategies for achieving the low-damage harvesting of shallots. Traditional rigid harvesting machinery not only consumes enormous energy but is also highly prone to inducing irreversible physical and mechanical damage.

To resolve this core conflict, a technological system centred on the “bionic drag reduction” of the front-end soil-engaging components and the “flexible enveloping” of the back-end clamping mechanisms has been constructed. In terms of drag reduction and detachment, by simulating the macroscopic geometric morphology and microscopic surface textures of soil animals, the cutting stress field can be effectively reconstructed, and capillary adhesion networks can be blocked, thereby substantially reducing the initial traction resistance. In terms of low-damage clamping, flexible actuation technology based on soft robotics has achieved highly conformable enveloping of the fragile pseudostems. The deep integration of the aforementioned “bionic drag reduction” and “flexible enveloping” technologies has laid a solid theoretical foundation for the mechanised harvesting of shallots and similar crisp and tender root crops.

### 5.2 Wear resistance bottlenecks of soft materials and adaptation challenges of rigid-flexible coupling

Although relevant technologies have demonstrated excellent low-damage potential in virtual simulations and controlled tests, they still face severe physical adaptation challenges during translation into actual field habitats. First, the issue of anti-fatigue wear under extreme working conditions is particularly prominent. High-humidity and heavy field soils are often mixed with high-hardness abrasive particles. During continuous operations, this is highly prone to causing micro-cutting damage to the bionic convex domes of soil-engaging components and the soft silicone actuators, resulting in the failure of detachment and air cushion effects, and a rebound in traction resistance. Secondly, there are limitations in the dynamic adaptation of rigid-flexible coupled systems. The low-frequency, large-amplitude bumping generated by the whole machine on undulating terrain is highly likely to breach the enveloping force boundaries, triggering secondary collision damage. Therefore, future equipment research and development must advance towards high-strength, wear-resistant composite materials and active vibration isolation suspension systems.

### 5.3 Trends in digital twin simulation and intelligent closed-loop control for shallot harvesting

With the cross-integration of computational mechanics and artificial intelligence technologies, the research and development paradigm for low-damage shallot harvesting equipment is accelerating its evolution towards “data-driven intelligent collaboration”. In the exploration of microscopic mechanical mechanisms, Multi-Body Dynamics and Discrete Element Method (MBD-DEM) coupling simulation technology has become an fundamental approach. In the future, it is important to construct high-fidelity digital twin models encompassing the “digging shovel-heavy soil-whole shallot plant” system. While MBD-DEM coupling provides excellent capabilities for simulating macroscopic kinematics and granular soil interactions, accurately resolving tissue-scale or cell-level stress concentrations within the tender pseudostems requires explicit coupling with Finite Element Method (FEM) models. Establishing such a multiscale MBD-DEM-FEM virtual environment will provide a comprehensive, non-destructive means of mechanistic analysis across multiple spatial scales.

At the level of intelligent perception and closed-loop regulation, vision systems based on deep learning and convolutional neural networks (CNNs) will endow harvesting equipment with precise field perception capabilities. However, it is essential to acknowledge the current limitations of digital twin simulations in precisely modelling the shallot digging and harvesting process. The primary bottleneck lies in the immense computational cost required to run real-time multiscale coupling algorithms. Furthermore, accurately calibrating the microscopic parameters of highly cohesive, high-humidity field soils intertwined with dense shallot root architectures remains a significant challenge. The dynamic fluid-structure interactions occurring when soft actuators deform against viscoelastic pseudostems also introduce non-linear variables that current simulation frameworks struggle to replicate with high precision. Overcoming these computational and calibration limitations is a prerequisite for deploying truly predictive digital twins in shallot harvesting. Deeply coupling visual feedforward data with force feedback sensors at the clamping end-effectors to construct a high-frequency response intelligent closed-loop control system, which dynamically adjusts the enveloping force of soft actuators in real time, will be the inevitable path to achieving highly robust and highly intact intelligent shallot harvesting.
